# Mechanistic Basis for the Differential Effects of Rivaroxaban and Apixaban on Global Tests of Coagulation

**DOI:** 10.1055/s-0038-1649507

**Published:** 2018-05-29

**Authors:** Paul Y. Kim, Calvin H. Yeh, Brian J. Dale, Beverly A. Leslie, Alan R. Stafford, James C. Fredenburgh, Jack Hirsh, Jeffrey I. Weitz

**Affiliations:** 1 Department of Medicine, McMaster University, Hamilton, Ontario, Canada; 2 Thrombosis and Atherosclerosis Research Institute, Hamilton, Ontario, Canada; 3 Department of Biochemistry and Biomedical Sciences, McMaster University, Hamilton, Ontario, Canada; 4 School of Pharmacy and Medical Sciences, University of South Australia, Adelaide, Australia

**Keywords:** DOAC, inhibition kinetics, coagulation assays, coagulation inhibitors

## Abstract

Rivaroxaban and apixaban are both small molecules that reversibly inhibit factor Xa. Compared with rivaroxaban, apixaban has minimal effects on the prothrombin time and activated partial thromboplastin time. To investigate this phenomenon, we used a factor Xa-directed substrate in a buffer system. Although rivaroxaban and apixaban inhibited factor Xa with similar K _i_ values at equilibrium, kinetic measurements revealed that rivaroxaban inhibited factor Xa up to 4-fold faster than apixaban ( *p* < 0.001). Using a discontinuous chromogenic assay to monitor thrombin production by prothrombinase in a purified system, rivaroxaban was 4-fold more potent than apixaban (K _i_ values of 0.7 ± 0.3 and 2.9 ± 0.5 nM, respectively; *p* = 0.02). Likewise, in thrombin generation assays in plasma, rivaroxaban prolonged the lag time and suppressed endogenous thrombin potential to a greater extent than apixaban. To characterize how the two inhibitors differ in recognizing factor Xa, inhibition of prothrombinase was monitored in real-time using a fluorescent probe for thrombin. The data were fit using a mixed-inhibition model and the individual association and dissociation rate constants were determined. The association rates for the binding of rivaroxaban to either free factor Xa or factor Xa incorporated into the prothrombinase complex were 10- and 1,193-fold faster than those for apixaban, respectively, whereas dissociation rates were about 3-fold faster. Collectively, these findings suggest that rivaroxaban and apixaban differ in their capacity to inhibit factor Xa and provide a plausible explanation for the observation that rivaroxaban has a greater effect on global tests of coagulation than apixaban.

## Introduction

Rivaroxaban and apixaban are oral factor Xa inhibitors that were developed as alternatives to warfarin for the prevention and treatment of arterial and venous thrombosis. 1 These direct, non–vitamin K antagonist oral anticoagulants (DOACs) are licensed in the United States and Europe for stroke prevention in patients with atrial fibrillation 2 3 and for the treatment of venous thromboembolism, 1 4 5 6 and have experienced rapid adoption in clinical practice. 7 8

The oral factor Xa inhibitors exhibit a similar mechanism of action. As small molecules that bind reversibly to the active site of factor Xa, rivaroxaban, and apixaban inhibit the enzyme with high affinity as evidenced by their sub-nanomolar inhibition constant (K _i_ ) values. 9 10 In addition to inhibiting free factor Xa, these agents inhibit factor Xa incorporated within prothrombinase, the complex of factor Xa and factor Va that assembles on activated platelets and converts prothrombin to thrombin. 9 10 Prothrombinase is the central effector of clotting because its assembly induces structural changes in factor Xa that increase the catalytic efficiency of prothrombin activation by over 100,000-fold. 11 Prothrombinase propagates coagulation by rapidly generating thrombin at sites of vascular injury. Therefore, the anticoagulant activity of rivaroxaban and apixaban reflects their rapid association with factor Xa incorporated within the prothrombinase complex.

Despite their similar affinities for factor Xa, rivaroxaban prolongs the prothrombin time (PT) and activated partial thromboplastin time (aPTT) more than apixaban. 12 13 14 15 16 17 18 A recent study by Jourdi et al reported that rivaroxaban binds free factor Xa with a 4-fold higher on-rate than apixaban and a 1.5-fold lower K _d_ , and modeling data suggested that this phenomenon explains the greater effect of rivaroxaban on the PT. 19 This difference is thought to contribute to the superiority of rivaroxaban in the thrombin generation assay. 19 20 However, the DOACs may have different inhibitory effects on factor Xa when it is incorporated into the prothrombinase complex and its substrate is prothrombin rather than a low-molecular-weight substrate. 9 10 Therefore, we compared rivaroxaban and apixaban in terms of their affinities for free factor Xa and for factor Xa incorporated into the prothrombinase complex and their rates of inhibition of prothrombinase-induced thrombin generation and we related these effects to those on the PT, aPTT, and thrombin generation assay.

## Materials and Methods

### Materials

Human prothrombin, factor Va, and dansylarginine-N-(3-ethyl-1,5-pentanediyl)amide (DAPA) were purchased from Haematologic Technologies (Essex Junction, Vermont, United States), whereas factor Xa and thrombin were purchased from Enzyme Research Laboratories (South Bend, IN). A variant prothrombin molecule cleaved solely at Arg320 (R155A, R271A, R284A, rMZ) was used to generate a stable recombinant form of meizothrombin, which was expressed in BHK cells and isolated as described previously. 21 The factor Xa-directed chromogenic substrate, Z-D-Arg-Gly-Arg- *p* -nitroaniline (S-2765), was purchased from Chromogenix (Milano, Italy), whereas the thrombin-directed substrate, Tos-Gly-Pro-Arg-pNA (Chromozym-thrombin [Chz-Th]), was from Hyphen BioMed (Neuville-sur-Oise, France). Fluorogenic factor Xa substrate Pefafluor Xa was from Pentapharm (Basel, Switzerland). Z-Gly-Gly-Arg-7-amino-4-methylcoumarin (GGR-AMC) and hirudin were from Bachem Bioscience, Inc. (Philadelphia, Pennsylvania, United States). Phospholipid vesicles were prepared in a 3:1 ratio of phosphatidylcholine and phosphatidylserine (PCPS) and stored in 10% sucrose at −80°C as described previously. 22 23 RecombiPlasTin 2G, which contains recombinant tissue factor at a concentration of 0.3 µg/mL, 24 was from Instrumentation Laboratory (Bedford, Massachusetts, United States). Prionex was from Pentapharm (Basel, Switzerland).

Rivaroxaban and apixaban, obtained from Suzhou Howsine Biological Technology Company (Suzhou, China), exhibited single and distinct peaks by HPLC analysis. After dissolving the agents in 100% dimethyl sulfoxide (DMSO) to a concentration of 10 mg/mL, they were stored in aliquots at −80°C. Molar concentrations were calculated using molecular weights of 435.9 and 459.5 for rivaroxaban and apixaban, respectively. Concentrations of rivaroxaban and apixaban were confirmed with the Rotochrom chromogenic anti-Xa assay (Diagnostica Stago, Parsippany, New Jersey, United States), which was performed using a ST4 coagulometer (Diagnostica Stago) with commercial rivaroxaban or apixaban calibrators (Technoclone, Vienna, Austria).

To prepare pooled normal human plasma (PNP), 50 mL of blood was collected from the antecubital veins of 10 to 15 healthy volunteers into a syringe containing 0.105 M sodium citrate. Platelet-poor plasma was obtained as described previously, with the exception that 2,500 × *g* was used for centrifugation. 24

### Prothrombin Time and Activated Partial Thromboplastin Time

Stock solutions of rivaroxaban and apixaban were diluted in PNP to 500 ng/mL (1,150 and 1,068 μM, respectively) and then serially diluted with PNP to a final concentration of 31.25 ng/mL (72 and 68 nM, respectively). The ranges of inhibitor concentrations were chosen to encompass the peak and trough plasma levels measured in patients treated with these agents. 25 26 The PT was performed using HemosIL HS PLUS (Instrumentation Laboratory, Lexington, Massachusetts, United States) on an ACL 7000 coagulometer (Instrumentation Laboratory) according to the manufacturer's instructions. The aPTT was performed using Actin FSL reagent (Siemens, Marburg, Germany) on a BCS XP analyzer (Siemens) according to the manufacturer's instructions. Assays were performed three times in duplicate at 37 **°** C. These PT and aPTT reagents were chosen because of their wide availability and their reported sensitivities to rivaroxaban and apixaban. 15 27

### Plasma-Based Thrombin Generation Assays

Thrombin generation assays were performed in 96-well microtiter plates at 37°C as previously described 22 24 28 using final concentrations of 3 pM tissue factor (HemosIL HS PLUS), 4 µM PCPS, 0.5 mM Z-Gly-Gly-Arg-AMC, and 7.5 mM CaCl _2_ , without added corn trypsin inhibitor. Studies were done in the absence or presence of rivaroxaban or apixaban at concentrations up to 2,300 and 2,120 µM (1,000 ng/mL), respectively. Assays were calibrated using Technothrombin TGA thrombin calibrators (Technoclone) according to the manufacturer's instructions with high and low thrombin concentration controls included in each run. The reactions were monitored over time using SpectraMax M5 fluorescence plate reader (Molecular Devices, Sunnyvale, California, United States) at excitation and emission at 360 and 460 nm, respectively, with the cutoff filter at 455 nm. All samples and controls were tested in quadruplicate.

### Determination of Inhibition Constants of Prothrombinase-Induced Thrombin Generation

To determine their inhibitory effects on prothrombinase-induced thrombin generation, rivaroxaban or apixaban was preincubated with prothrombin at 25 **°** C prior to addition to a solution containing factor Xa, factor Va, PCPS, and calcium. Reactions were performed in clear or black flat-bottom 96-well polystyrene plates (Corning Inc., Corning, New York, United States). The final activation solution consisted of 20 µL of 0 to 200 nM inhibitor, 0.5 nM factor Xa, 5.5 nM factor Va, 50 µM PCPS, 500 nM prothrombin, and 5 mM CaCl _2_ in 20 mM HEPES, 150 mM NaCl, pH 7.4 (HBS) containing 0.01% Tween-80 (HBST). Reactions were stopped after 20 seconds by addition of 20 µL of 10 mM EDTA, and thrombin activity was measured by adding 60 µL of 500 µM Chz-Th and monitoring its hydrolysis at 405 nm using a plate reader. The rates of Chz-Th hydrolysis were converted to concentrations of thrombin generated using a specific activity of 17.2 mOD·min ^−1^ ·nM ^−1^ , which was determined in a separate experiment. Thrombin concentrations were divided by reaction time to obtain the rate of thrombin formation (nM/s) and divided by the factor Xa concentration to obtain the turnover number (k _cat_ ). The turnover number was then plotted against the concentration of inhibitor, and the data were fit to a rectangular hyperbola equation by nonlinear regression analysis using TableCurve version 4.04 (Jandel Scientific):

V = V _0_ + (V _min_ [I]) / (IC _50_ + [I])

where V _0_ and V _min_ are the turnover numbers of thrombin generation in the absence and presence of saturating concentrations of inhibitor, respectively; IC _50_ is the concentration of inhibitor required to produce half-maximal reduction; and [I] is the inhibitor concentration. K _i_ values were then obtained from the IC _50_ value using the Cheng–Prusoff equation:

IC _50_ = [1 + ([S] / K _M_ )] K _i_

where [S] is the prothrombin concentration and K _M_ is the Michaelis constant of prothrombinase for prothrombin (157.4 nM) determined in a separate experiment as described previously. 21

### Determination of Steady-State Inhibition Constants of Factor Xa

To measure the affinity of the inhibitors for factor Xa at equilibrium, 10 µL aliquots containing 0 to 200 nM rivaroxaban or apixaban were added to wells containing 10 µL of 50 nM factor Xa. Residual factor Xa activity was then measured by adding S-2765 and CaCl _2_ to a final volume of 100 µL, yielding final concentrations of 400 µM and 5 mM, respectively. Absorbance was monitored at 405 nm using a SpectraMax Plus plate reader (Molecular Devices, Sunnyvale, California, United States) and the rates of S-2765 hydrolysis were converted to factor Xa concentrations using a specific activity of 25.7 mOD·min ^−1^ ·nM ^−1^ , which was determined in a separate experiment (not shown). Residual concentrations of factor Xa were then plotted against anticoagulant concentrations, and the data were analyzed as above to obtain the K _i_ values. All inhibition experiments were conducted at 25°C in HBST containing 0.1% Prionex and 5 mM CaCl _2_ (HBSTC). Stock solutions of inhibitors were diluted in HBST, such that the final concentration of DMSO was < 0.01% (v/v); a concentration that, in control experiments, did not influence the hydrolysis of S-2765 by factor Xa.

### Rate Constants of Inhibition of Factor Xa

The rates of association and dissociation of the inhibitors with factor Xa were determined simultaneously in HBSTC buffer under pseudo first-order conditions using Molecular Devices Flex Station 3 plate reader. In microplate wells, 100-µL aliquots of a solution containing the inhibitor and Pefafluor Xa were added to 100-µL aliquots of factor Xa, such that final concentrations were 2.5 nM, 100 µM, and 0.25 nM, respectively. Samples were rapidly mixed by the instrument and fluorescence was monitored at 2-second intervals at 37°C with excitation and emission wavelengths of 342 and 440 nm, respectively, and a 435-nm cutoff filter. To convert relative fluorescence units to nominal concentrations of 7-amino 4-methylcoumarin (AMC), a standard curve was generated from intensity values at 440 nm (I _440_ ) of varying concentrations of fully hydrolyzed Pefafluor Xa.

**Scheme 1:**
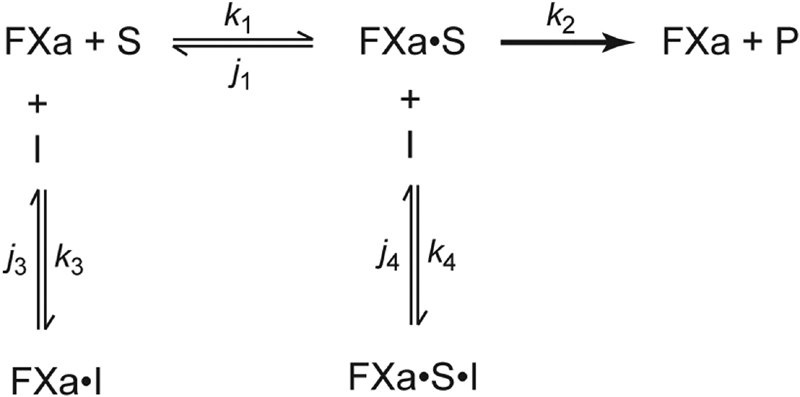
Mixed inhibition of factor Xa by apixaban or rivaroxaban. This model depicts two ways in which factor Xa may bind its inhibitors. FXa, factor Xa; S, substrate (Pefafluor Xa); I, inhibitor (apixaban or rivaroxaban).

For analysis of the results, a mixed inhibition model 10 was fit to each time course of AMC production ( Scheme 1 ) using equations (see Supplementary Materials ) formulated to determine the best-fit individual rate constants of association and dissociation by numerical integration using Berkeley Madonna Software (University of California, Berkeley, California, United States). 21 In this model, FXa is the free factor Xa concentration, S is the concentration of Pefafluor Xa, I is the concentration of inhibitor (apixaban or rivaroxaban), FXa·S is the factor Xa-Pefafluor Xa complex, FXa·I is the factor Xa–inhibitor complex, and FXa·S·I is the ternary factor Xa–Pefafluor Xa–inhibitor complex. The best-fit rate constants were first determined in the absence of inhibitors, then the theoretical dissociation constants for each inhibitor were calculated for free factor Xa (K _Xa1_ = *j*_3_ / *k*_3_ ) and for the factor Xa–substrate complex (K _Xa2_ = *j*_4_ / *k*_4_ ).

### Rate Constants of Inhibition of Factor Xa in Prothrombinase

The individual rate constants for association and dissociation of the inhibitors with factor Xa incorporated into the prothrombinase complex were determined by measuring thrombin generation to infer residual factor Xa activity in real time. Thrombin generation was quantified using DAPA, a probe that specifically inhibits thrombin and exhibits increased fluorescence on doing so. 29 For the prothrombinase reaction, 60 µL of a 2× stock solution (solution A) containing 1 nM factor Xa, 11 nM factor Va, 50 µM PCPS, and 10 µM DAPA was added to wells of a 96-well UV-compatible microtiter plate (Corning). In a separate low protein-binding 96-well plate (Thermo), 80 µL of a 2× solution (solution B) containing 1 µM prothrombin and varying concentrations of either apixaban or rivaroxaban (0, 2, 10, or 30 nM) was added. All reagents were dissolved in 20 mM Tris-HCl, 0.15 M NaCl, pH 7.4, and 5 mM CaCl _2_ . Both plates were then placed in a FlexStation 3 (Molecular Devices) and allowed to equilibrate at 37°C for 5 minutes and fluorescence was monitored by reading from the bottom of the wells at 2-second intervals using excitation and emission wavelengths of 280 and 545 nm, respectively, and a 530 nm cutoff filter. After determining baseline fluorescence for 30 seconds, 60-µL aliquots of solution B were then transferred to the respective wells of the first plate, and samples were mixed by instrument trituration. Experiments were performed at least three times. To convert the fluorescence signal to nominal thrombin concentration, a standard curve was generated using known amounts of thrombin in the presence of DAPA.

**Scheme 2:**
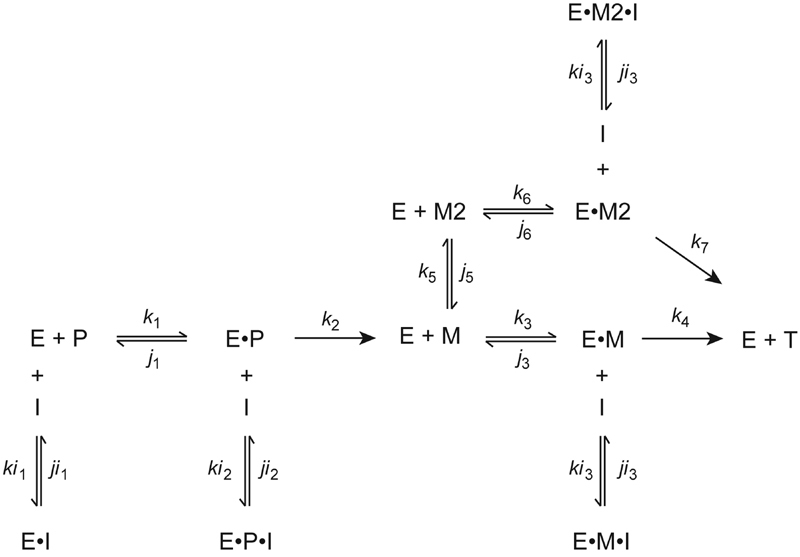
Mixed inhibition of factor Xa in prothrombinase by apixaban or rivaroxaban during prothrombin activation. This model depicts three ways in which prothrombinase may bind its inhibitors. Ratcheting is included in this model, which signifies a conformational change of the intermediate, meizothrombin, so that it is able to be further processed to thrombin. E, prothrombinase; P, prothrombin; M, meizothrombin; M2, ratcheted meizothrombin; T, thrombin; I, inhibitors (apixaban or rivaroxaban).

For analysis, data were fit using a mixed inhibition model 10 ( Scheme 2 ), wherein all forms of factor Xa (E), both free or within prothrombinase, could interact with the inhibitor (I). Prothrombin (P) activation by prothrombinase was limited to the meizothrombin (M) pathway. This approach is justified because (1) meizothrombin is the predominant intermediate generated when prothrombin is activated by factor Xa in the presence of factor Va; (2) there was minimal prethrombin-2 accumulation by SDS-PAGE analysis (data not shown); and (3) unlike meizothrombin or thrombin, prethrombin-2 does not possess a functional active site. 30 Therefore, the change in fluorescence signal can be attributed to thrombin or meizothrombin. Ratcheting of meizothrombin, a phenomenon whereby meizothrombin alters its conformation and renders the second cleavage by prothrombinase more effectively, was included ( *k*_5_ , *j*_5_ in Scheme 2 ) to be consistent with current models of prothrombinase. 31 The inhibition rate constants for the binding of the inhibitors to prothrombinase when it is in complex with either form of meizothrombin were kept the same. In addition, the fluorescence quantum yield of meizothrombin was taken to be 1.5 times that of thrombin. 32 33 Based on this model, 13 differential equations for the reactants and product were formulated (see Supplementary Materials ) to determine the best-fit rate constants as described earlier. Briefly, the rate constants of the main reaction that do not involve the inhibitor ( *k*_x_ and *j*_x_ ) were determined first using similar step-wise restrictions imposed over rounds of fitting ( Supplementary Table S1 ). Once the best-fit *k*_x_ and *j*_x_ values for each set of data were obtained, they were fixed in order to determine the best-fit *ki*_x_ and *ji*_x_ values for each inhibitor. The theoretical dissociation constants for each inhibitor were then calculated for prothrombinase without prothrombin (K _Pase1_ = *ji*_1_ / *ki*_1_ ), prothrombinase in complex with prothrombin (K _Pase2_ = *ji*_2_ / *ki*_2_ ), and for prothrombinase in complex with either form of meizothrombin (K _Pase3_ = *ji*_3_ / *ki*_3_ ) using the individual on- and off-rates.

To determine whether the divergent effects of rivaroxaban and apixaban on the coagulation assays are due to differential inhibition of factor Xa leading to differences in substrate recognition, a mutated form of prothrombin rMZ, which can only be cleaved at Arg320, was used as the substrate so that initial cleavage at this site could be examined in isolation. 34 rMZ activation by prothrombinase was monitored and the data were analyzed under conditions identical to those used with wild-type prothrombin. The model shown in Scheme 1 was used to describe this reaction, where the substrate (S) was replaced with rMZ and the product (P) was replaced with rMZa. The theoretical dissociation constants for rivaroxaban or apixaban were again calculated using the best-fit rate constants determined as described earlier.

### Statistical Analyses

All experiments were performed at least in triplicate and data are expressed as mean ± SD. Inhibition constants (K _i_ ) and inhibition rates ( *k*_2_ ) for rivaroxaban and apixaban were compared by Student's *t* -tests using Microsoft Office Excel 2010. PT, aPTT, and thrombin generation data were compared by two-way analysis of variance using GraphPad Prism 6 (San Diego, California, United States). For all analyses, *p* -values < 0.05 were considered statistically significant.

## Results

### Effect of Inhibitors on the PT and aPTT

**Fig. 1:**
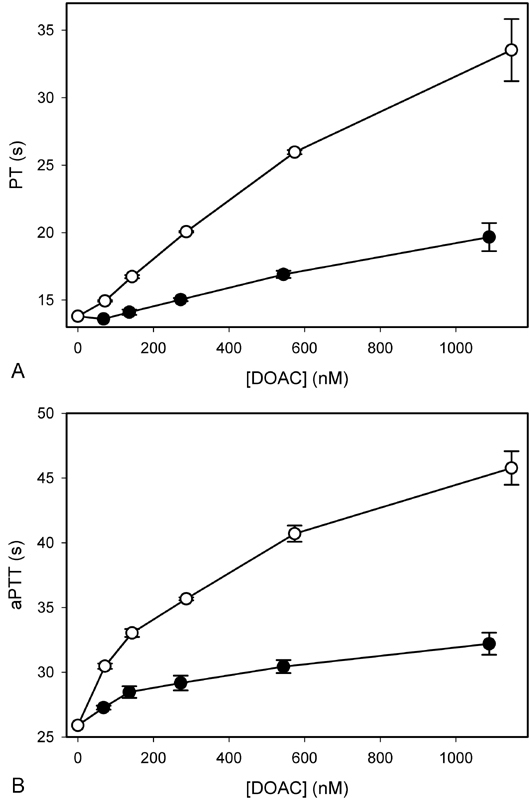
Comparison of the effects of rivaroxaban and apixaban on the prothrombin time (PT) and activated partial thromboplastin time (aPTT). The ( **A** ) PT and ( **B** ) aPTT were determined in human plasma in the presence of increasing concentrations of apixaban ( *closed* ) or rivaroxaban ( *open* ). Symbols reflect the mean ± SD of four experiments.

The effects of rivaroxaban and apixaban on the PT and aPTT were examined using concentrations up to 2,530 and 2,350 µM (1,100 ng/mL), respectively, well beyond the peak plasma levels obtained with the doses of the drugs that are used clinically. 26 35 Although both agents prolonged the PT and aPTT in a concentration-dependent manner ( Fig. 1 ), rivaroxaban had a significantly ( *p* < 0.0001) greater effect on both tests than apixaban, consistent with previous reports. 13 27 Therefore, rivaroxaban has a greater effect on global tests of coagulation than apixaban, regardless of whether coagulation is activated via the extrinsic or intrinsic pathway.

### Plasma-Based Thrombin Generation Assay

**Fig. 2:**
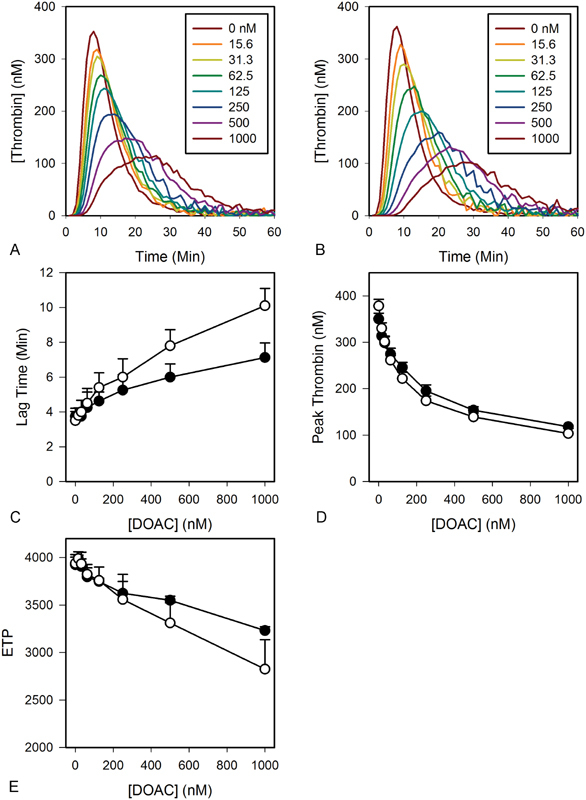
Comparison of the effects of apixaban and rivaroxaban on thrombin generation in plasma. Thrombin generation in plasma containing 4 µM PCPS was triggered by addition of 3 pM tissue factor and 7.5 mM CaCl _2_ and quantified by monitoring the hydrolysis of 0.5 mM Z-Gly-Gly-Arg-AMC in the absence or presence of ( **A** ) apixaban, or ( **B** ) rivaroxaban, at the indicated concentrations. Using the thrombin generation profiles, ( **C** ) lag time, ( **D** ) peak thrombin concentration, and ( **E** ) ETP are measured and plotted *versus* the concentrations of apixaban ( *closed* ) or rivaroxaban ( *open* ). Data reflect the mean ± SD of three experiments. ETP, endogenous thrombin potential.

The inhibitory effects of rivaroxaban and apixaban on thrombin generation induced by 3 pM tissue factor in plasma were compared using a calibrated thrombin generation assay. Both agents prolonged the lag time, reduced peak thrombin concentration, and decreased endogenous thrombin potential (ETP) in a concentration-dependent manner ( Fig. 2 ). Rivaroxaban significantly prolonged the lag time and decreased ETP compared with apixaban, consistent with previous reports. 19 20 The agents had similar effects on other parameters of thrombin generation.

### Inhibition of Factor Xa

**Fig. 3:**
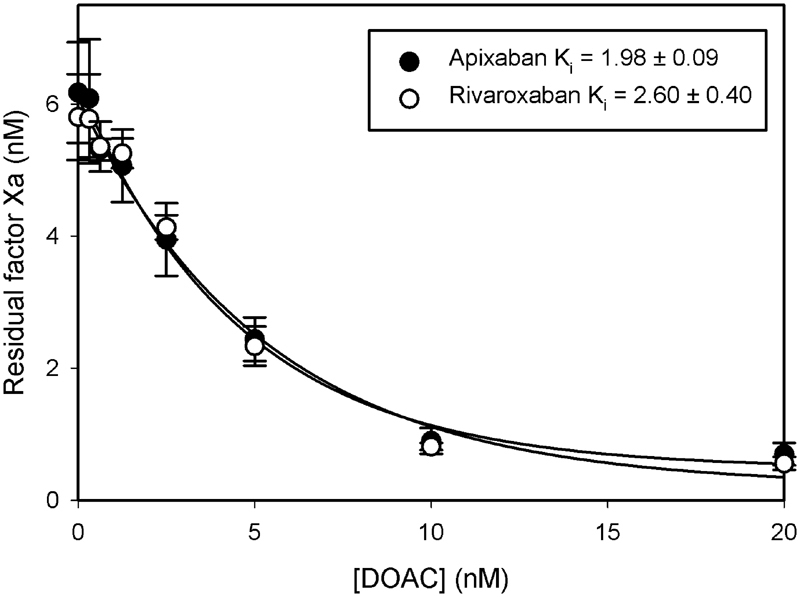
Comparison of the effects of rivaroxaban and apixaban on factor Xa chromogenic activity. Inhibition constants (K _i_ ) were determined by incubating 5 nM factor Xa with rivaroxaban ( *open* ) or apixaban ( *closed* ) at the indicated concentrations, and residual active factor Xa concentration was determined by monitoring hydrolysis of 500 μM S-2765. Symbols reflect the mean ± SD of three to four experiments. Lines are fit to a rectangular hyperbola equation by nonlinear regression analysis.

To begin to investigate the mechanism responsible for the divergent effects of rivaroxaban and apixaban, we first measured their steady-state binding affinities for free factor Xa and for factor Xa assembled in the prothrombinase complex using a chromogenic assay for factor Xa. Rivaroxaban and apixaban inhibited the chromogenic activity of free factor Xa in a concentration-dependent manner ( Fig. 3 ) with K _i_ values of 2.6 ± 0.4 nM and 2.0 ± 0.1 nM, respectively, values comparable with those previously reported. 9 10 At a concentration of 20 nM, both agents inhibited the chromogenic activity of 5 nM factor Xa by ∼85%.

Studies of factor Xa inhibition were then repeated in the presence of factor Va, PCPS, and prothrombin. Hirudin was added to prevent substrate hydrolysis by the generated thrombin. As observed with factor Xa alone, in the presence of prothrombinase components, rivaroxaban and apixaban inhibited over 95% of factor Xa chromogenic activity in the same dose-dependent manner (not shown) with K _i_ values of 1.0 ± 0.6 nM and 0.9 ± 0.8 nM, respectively, values not significantly different from those determined with free factor Xa. Together, these data confirm that rivaroxaban and apixaban bind to free factor Xa and factor Xa incorporated into the prothrombinase complex with comparable affinities. 9 10 Therefore, differences in steady-state binding affinities do not explain the divergent effects of rivaroxaban and apixaban on the PT, aPTT, and thrombin generation.

### Inhibition of Prothrombinase-Induced Thrombin Generation

**Fig. 4:**
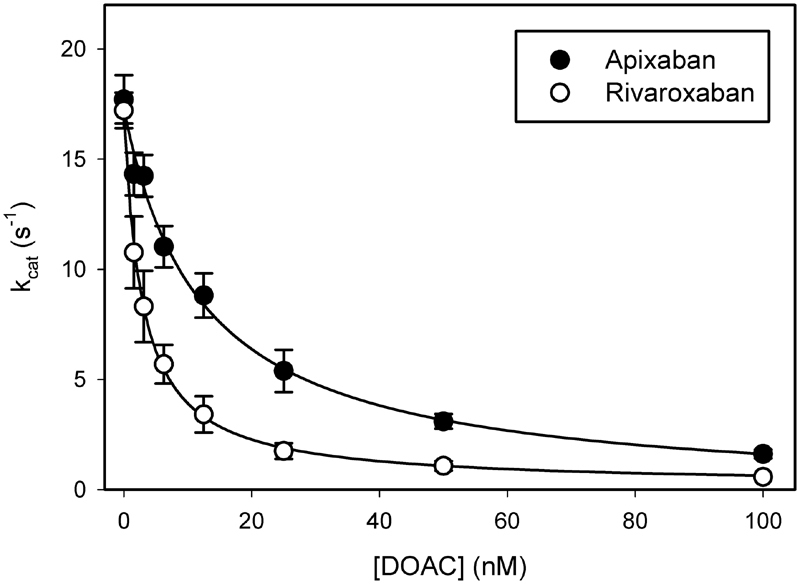
Comparison of the effects of rivaroxaban and apixaban on prothrombin activation by prothrombinase. Inhibition constants (K _i_ ) were determined by incubating increasing concentrations of rivaroxaban ( *open* ) or apixaban ( *closed* ) with 0.5 nM factor Xa, 5.5 nM factor Va, 50 µM PCPS, 500 nM prothrombin, and 5 mM CaCl _2_ . After 20 s, reactions were stopped by addition of EDTA to 10 mM and generated thrombin was quantified by chromogenic assay with Chz-Th. Rates of prothrombin activation are plotted versus inhibitor concentration. Symbols reflect the mean ± SD of three to four experiments. Lines are fit to a rectangular hyperbola equation by nonlinear regression analysis.

The previous experiments examined the effect of the inhibitors on factor Xa chromogenic activity, an interaction that excludes those employed by prothrombin at sites remote from the active site. 36 To examine the effect of the inhibitors when prothrombin is the substrate of prothrombinase, we quantified thrombin generation. Although both inhibitors reduced prothrombinase-induced thrombin production in a concentration-dependent manner ( Fig. 4 ), the K _i_ value for rivaroxaban was 4-fold lower than that for apixaban (0.7 ± 0.3 nM and 2.9 ± 0.5 nM, respectively; *p* < 0.02). Therefore, rivaroxaban is a more potent inhibitor of prothrombinase-induced thrombin formation than apixaban.

### Inhibition Rate Constants: Factor Xa Binding

**Fig. 5:**
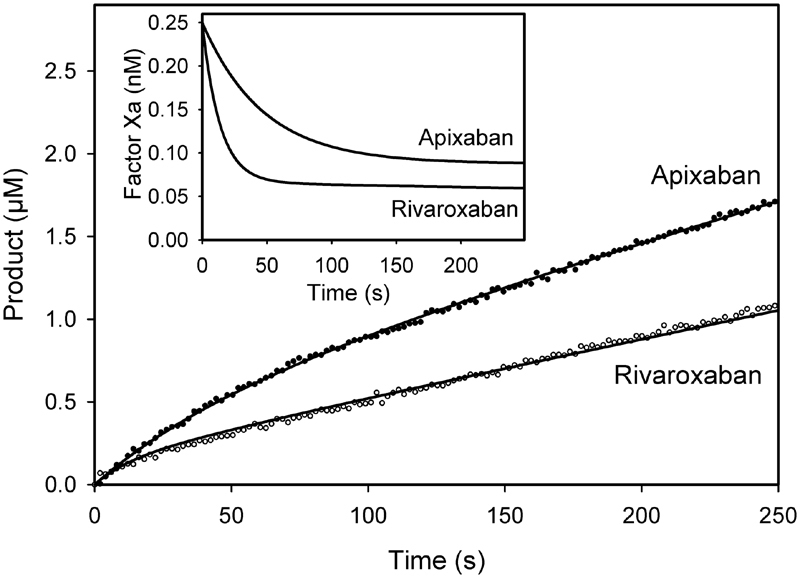
Inhibition of factor Xa amidolytic activity in the presence of apixaban and rivaroxaban. Cleavage of 100 µM Pefafluor Xa by 0.25 nM factor Xa was monitored in a fluorescent plate reader at 2-s intervals. Reactions were performed at 37°C in HBSTC containing 2.5 nM apixaban ( *closed* ) or rivaroxaban ( *open* ). Fluorescence intensity values were converted to concentration of 4-amino methyl coumarin (product) and plotted *versus* time. Data were fit to a mixed inhibition model (lines). The inset shows nominal residual factor Xa concentration determined from the fitted data plotted *versus* time.

To better understand why rivaroxaban and apixaban have different effects on prothrombinase-induced thrombin generation, we next compared the binding kinetics of the inhibitors with factor Xa using a mixed inhibition model ( Scheme 1 ). 10 Inhibition was quantified by continuously monitoring factor Xa fluorogenic activity in the presence of rivaroxaban or apixaban. Although the time courses revealed gradual reduction of factor Xa fluorogenic activity, as evidenced by declining rates of fluorophore release with both inhibitors ( Fig. 5 ), plots of residual factor Xa activity revealed more rapid inhibition with rivaroxaban than with apixaban ( Fig. 5 , *inset* ). Plots of product *versus* time were analyzed by global fitting and the best-fit values for *k*_1_ , *j*_1_ , and *k*_2_ were determined to be 7.05 ± 0.30 nM ^−1^ ·s ^−1^ , 1.74 ± 0.09 s ^−1^ , and 187 ± 14 s ^−1^ , respectively. With these values fixed, the best-fit values for the rate constants for inhibition were determined ( Table 1 ). The on- and off-rate constants for binding factor Xa alone ( *k*_3_ and *j*_3_ , respectively) for rivaroxaban were, on average, 21-fold faster than those for apixaban. A similar trend was observed for inhibitor binding to the factor Xa–fluorogenic substrate complex ( *k*_4_ and *j*_4_ ), whereby rivaroxaban bound, on average, 25-fold faster than apixaban. Therefore, contrary to a previous report, 19 our data suggest that a faster on-rate is not the sole explanation for the greater effect of rivaroxaban on tests of coagulation than apixaban.

**Table 1:** Kinetic parameters of factor Xa inhibition by apixaban and rivaroxaban determined according to Scheme 1 by nonlinear regression analyses of chromogenic substrate cleavage data

Constant	Inhibitor		Fold
	Apixaban	Rivaroxaban	
*k*_3_ (nM ^−1^ ·s ^−1^ )	(3.65 ± 0.24) × 10 ^−3^	(3.77 ± 0.32) × 10 ^−2^	10.3
*j*_3_ (s ^−1^ )	(1.04 ± 0.06) × 10 ^−3^	(3.21 ± 0.28) × 10 ^−2^	30.9
K _Xa1_ (nM)	(2.85 ± 0.23) × 10 ^−1^	(8.54 ± 0.78) × 10 ^−1^	3.0
*k*_4_ (nM ^−1^ ·s ^−1^ )	(2.95 ± 0.29) × 10 ^−4^	(1.34 ± 0.04) × 10 ^−2^	45.4
*j*_4_ (s ^−1^ )	(3.86 ± 0.32) × 10 ^−3^	(2.01 ± 0.17) × 10 ^−2^	5.2
K _Xa2_ (nM)	(1.32 ± 0.15) × 10 ^1^	1.49 ± 0.12	0.1

Notes: K _Xa_ values represent off- and on-rate quotients. Fold is the ratio between rivaroxaban and apixaban.

### Inhibition Rate Constants: Prothrombin Activation

**Fig. 6:**
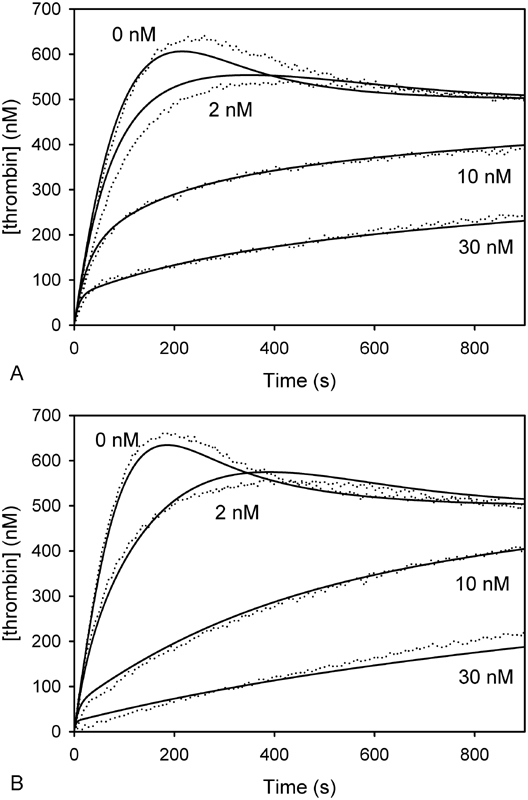
Inhibition of factor Xa assembled in the prothrombinase complex by the presence of apixaban and rivaroxaban. Activation of 500 nM prothrombin by 0.5 nM prothrombinase (0.5 nM factor Xa, 5 nM factor Va, 50 µM PCPS, 5 mM CaCl _2_ ) containing ( **A** ) apixaban or ( **B** ) rivaroxaban at varying concentrations (2, 10, or 30 nM) was monitored using DAPA in a fluorescent plate reader at 37°C. Thrombin generation was monitored at 2-s intervals. Data were fit to a mixed inhibition model (lines).

Because of the differences observed in the prothrombinase endpoint assay ( Fig. 4 ), we used a continuous assay to determine the kinetic rate constants for inhibition of factor Xa assembled in prothrombinase. Thrombin generation was quantified by continuously monitoring the increase in fluorescence that accompanies binding of DAPA, a fluorophore that binds exclusively to the active site of meizothrombin or thrombin. 29 Plots of thrombin concentration *versus* time in the presence of varying concentrations of the inhibitors were analyzed using the mixed inhibition model ( Scheme 2 ), wherein the inhibitor is allowed to bind to prothrombinase-bound factor Xa in the absence or presence of prothrombin or meizothrombin. Concentrations of thrombin and meizothrombin over time were calculated by numerical integration. Individual rate constant parameter values were optimized using the Simplex procedure 37 as described previously. 21 Best-fit association and dissociation rate constants, and the calculated K _Pase_ value, representing the dissociation constant for inhibitor with factor Xa in prothrombinase, are illustrated in Table 2 . The rate constants associated with prothrombin activation by prothrombinase in the absence of inhibitor are shown in Supplementary Table S1 . Using the best-fit rate constants, regression lines for the progress curves in the presence of 2 to 30 nM apixaban ( Fig. 6A ) or rivaroxaban ( Fig. 6B ) were generated. On-rate constants *ki*_1_ and *ki*_2_ for rivaroxaban were 1,193-fold and 3-fold higher than those for apixaban, respectively ( Table 2 ). The higher *ki*_1_ and *ki*_2_ values with rivaroxaban are comparable with the 10-fold higher *k*_3_ and 45-fold higher *k*_4_ values observed with rivaroxaban relative to apixaban in the factor Xa chromogenic assay ( Table 1 ). Interestingly, the on-rate for rivaroxaban with the prothrombinase–meizothrombin complex, *ki*_3_ , was 3-fold slower than that for apixaban. Therefore, rivaroxaban and apixaban have different off-rates as well as different on-rates. The greatest difference in off:on ( *ji*_x_ / *ki*_x_ ) rate ratios of these constants (K _Pase_ ) was with free enzyme inhibition (K _Pase1_ ), where the value for rivaroxaban was 454-fold lower than that for apixaban, mostly due to the very low *ki*_1_ value for apixaban ( Table 2 ). Closer inspection reveals that only the on-rate of apixaban is altered by the assembly of factor Xa into prothrombinase, suggesting that rivaroxaban and apixaban interact with factor Xa differently.

**Table 2:** Kinetic parameters of inhibition of factor Xa incorporated in prothrombinase by apixaban or rivaroxaban as measured using thrombin-DAPA fluorescence

Reaction	Constant	Inhibitor		Fold
		Apixaban	Rivaroxaban	
E + I ⇔ E · I	*ki*_1_ (nM ^−1^ ·s ^−1^ )	(4.51 ± 0.02) × 10 ^−6^	(5.38 ± 0.35) × 10 ^−3^	1192.9
	*ji*_1_ (s ^−1^ )	(4.99 ± 0.05) × 10 ^−4^	(1.31 ± 0.11) × 10 ^−3^	2.6
	K _Pase1_ (nM)	(1.11 ± 0.02) × 10 ^2^	(2.44 ± 0.31) × 10 ^−1^	0.002
E · P + I ⇔ E · P · I	*ki*_2_ (nM ^−1^ ·s ^−1^ )	(4.30 ± 0.18) × 10 ^−3^	(1.24 ± 0.02) × 10 ^−2^	2.9
	*ji*_2_ (s ^−1^ )	(5.81 ± 0.30) × 10 ^−3^	(1.12 ± 0.15) × 10 ^−2^	1.9
	K _Pase2_ (nM)	1.35 ± 0.05	(8.99 ± 1.16) × 10 ^−1^	0.7
E · M + I ⇔ E · M · I or E · M2 + I ⇔ E · M2 · I	*ki*_3_ (nM ^−1^ ·s ^−1^ )	(6.20 ± 0.33) × 10 ^−3^	(2.37 ± 0.22) × 10 ^−3^	0.4
	*ji*_3_ (s ^−1^ )	(2.64 ± 0.19) × 10 ^−5^	(2.95 ± 0.11) × 10 ^−3^	111.7
	K _Pase3_ (nM)	(4.28 ± 0.01) × 10 ^−1^	1.25 ± 0.16	2.9

Notes: K _Pase_ values represent off- and on-rate quotients. Fold is the ratio between rivaroxaban and apixaban.

### Inhibition Rate Constants: rMZ Activation

**Fig. 7:**
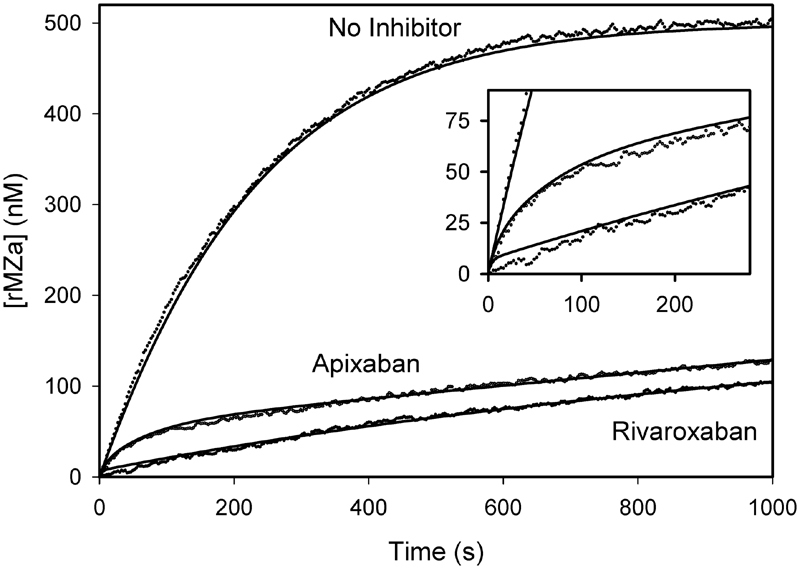
rMZ was used instead of prothrombin to monitor activation cleavage at Arg320 by prothrombinase using DAPA in the absence or presence of apixaban or rivaroxaban (30 nM). rMZa generation was monitored at 2-s intervals in a fluorescent plate reader at 37°C. Data were fit to a mixed inhibition model (lines).

Because prothrombin activation requires two cleavages, kinetic analyses are complicated by the requirement to account for parallel reaction pathways. To simplify the analysis, we used rMZ, a recombinant prothrombin derivative that can only be cleaved at Arg320. 34 Activation of rMZ was monitored using DAPA and kinetic analyses were performed as outlined earlier. Best-fit rate constants ( Table 3 ) were used to generate regression lines ( Fig. 7 ). The off:on ( *ji*_x_ / *ki*_x_ ) ratio of free prothrombinase (K _Pase1_ ) was 16-fold lower with rivaroxaban than with apixaban, whereas K _Pase2_ , representing inhibition of the prothrombinase-rMZ complex, was 3-fold lower with rivaroxaban than with apixaban. The difference in the effect between rivaroxaban and apixaban on rMZ activation is most evident in the initial stages of rMZ activation ( Fig. 7 , inset), similar to what was observed with wild-type prothrombin ( Fig. 6 ). Therefore, these data suggest that rivaroxaban and apixaban have different effects on the initial interaction of factor Xa in the prothrombinase complex with prothrombin, and that this difference occurs upon the initial presentation and cleavage of prothrombin at Arg320.

**Table 3:** Kinetic parameters of factor Xa inhibition by apixaban or rivaroxaban as measured using meizothrombin-DAPA fluorescence

Reaction	Constant	Inhibitor		Fold
		Apixaban	Rivaroxaban	
E + I ⇔ E · I	*ki*_1_ (nM ^−1^ ·s ^−1^ )	(4.70 ± 0.39) × 10 ^−3^	(3.46 ± 0.49) × 10 ^−2^	7.4
	*ji*_1_ (s ^−1^ )	(4.74 ± 0.23) × 10 ^−2^	(2.15 ± 0.18) × 10 ^−3^	0.05
	K _Pase1_ (nM)	(1.01 ± 0.12) × 10 ^1^	(6.24 ± 0.41) × 10 ^−2^	0.006
E · rMZ + I ⇔ E · rMZ · I	*ki*_2_ (nM ^−1^ ·s ^−1^ )	(1.88 ± 0.14) × 10 ^−2^	(6.41 ± 0.44) × 10 ^−3^	0.3
	*ji*_2_ (s ^−1^ )	(1.02 ± 0.10) × 10 ^−3^	(1.23 ± 0.08) × 10 ^−4^	0.1
	K _Pase2_ (nM)	(5.44 ± 0.42) × 10 ^−2^	(1.93 ± 0.25) × 10 ^−2^	0.4

Notes: K _Pase_ values represent off- and on-rate quotients. Fold is the ratio between rivaroxaban and apixaban.

## Discussion

Previous studies have shown that rivaroxaban has a greater effect on the PT and aPTT than on apixaban. 12 13 14 17 To determine whether the differences between the on-rates of the inhibitors for free factor Xa 19 are maintained when factor Xa is incorporated into prothrombinase and to correlate those with global tests of coagulation, we compared the effects of rivaroxaban and apixaban in three different systems. First, we compared their effects in plasma on the PT, aPTT, and thrombin generation assay. Second, we determined their K _i_ values for free factor Xa and factor Xa incorporated into prothrombinase. Third, we measured the rate constants for inhibition of factor Xa alone and when assembled in prothrombinase.

As previously reported, rivaroxaban and apixaban inhibited factor Xa with similar K _i_ values, confirming that the agents bind factor Xa with similar affinities at equilibrium. 9 10 19 Also consistent with previous results, at equimolar concentrations, rivaroxaban prolonged the PT and aPTT more than apixaban. 12 13 14 In the thrombin generation assay, rivaroxaban and apixaban influenced all parameters in a concentration-dependent manner, although rivaroxaban prolonged the lag time and reduced ETP to a significantly greater extent than apixaban. Because clot formation typically occurs shortly after the lag time, the bulk of thrombin is generated after the clot has formed. The fact that rivaroxaban prolongs the lag time more than apixaban is consistent with its greater effects in clot-based assays such as PT and aPTT.

The effects of apixaban and rivaroxaban on the rates of prothrombin activation were examined. These experiments differ from those for determination of K _i_ because all the components of prothrombinase are present, and we quantified thrombin generation rather than factor Xa activity. 38 Rivaroxaban was 2-fold more potent than apixaban at inhibiting prothrombinase-induced thrombin generation. This was further investigated by determining the association and dissociation rate constants for inhibition of factor Xa. Using a mixed-type inhibition model, on- and off-rates for rivaroxaban were higher than those for apixaban. These findings are consistent with the results from the prothrombinase and clotting assays and they suggest that there are mechanistic differences in the way that rivaroxaban and apixaban interact with factor Xa incorporated within the prothrombinase complex.

rMZ was used to shed further light on the molecular mechanism responsible for the divergent inhibitory effects of rivaroxaban and apixaban on factor Xa incorporated into the prothrombinase complex. Like the results with native prothrombin, rivaroxaban is more potent than apixaban at inhibiting prothrombinase-induced activation of rMZ ( Figs. 6 and 7 ). Together, these data suggest that the differences between the inhibitors can mainly be attributed to their divergent inhibitory effects on the initial activation cleavage of prothrombin at Arg320 by factor Xa, which is the major pathway of prothrombin activation in the presence of factor Va, and the only available cleavage site in rMZ. The off:on ratios for rivaroxaban were 1.5-fold ( Table 2 ) and 2.8-fold ( Table 3 ) lower than those for apixaban with native prothrombin and rMZ, respectively. Interestingly, incorporation of factor Xa into prothrombinase appears to have more profound effects on the inhibitory activity of apixaban than rivaroxaban, suggesting that there may be fundamental differences in the way that the two agents interact with factor Xa when it assembles into its activation complex.

A recent study suggested that the differential effects of rivaroxaban and apixaban on the PT reflected a 4-fold faster association rate constant of rivaroxaban for free factor Xa. 19 We have extended this concept by examining factor Xa assembled into the prothrombinase complex. Thus, we have shown that the association rate of rivaroxaban is faster than that of apixaban not only with free factor Xa, but also with factor Xa assembled into the prothrombinase complex, and with prothrombinase activation of prothrombin. Furthermore, using the rMZ variant, we demonstrate that the initial cleavage of prothrombin at Arg320 is faster with rivaroxaban than with apixaban. Biochemical modeling of enzyme–substrate–inhibitor interaction confirms that the difference between the inhibitors is not limited to the association rates with the enzyme (either factor Xa or prothrombinase). Instead, differences in both the association and dissociation rate constants are responsible for the greater activity of rivaroxaban relative to apixaban.

Although rivaroxaban and apixaban were both developed as small molecule factor Xa inhibitors, differences in their kinetic parameters and functional potencies are evident. This could arise from differences in sites of contact with the enzyme, as well as how the substrate binds. Examination of the crystal structure of rivaroxaban and apixaban in complex with factor Xa reveals that although both factor Xa inhibitors are similarly L-shaped, 39 they exhibit distinct interactions with substrate-binding subsites S1 and S4 within the active site cleft and differ in rigidity. 40 41 42 43 These differences may account for the disparate association rate constants and their divergent effects on prothrombin activation.

Our study has some limitations. First, the kinetic analyses were conducted in purified systems, whereas the clotting and thrombin generation assay were performed in plasma. Therefore, translation of findings from one system to the other can be problematic. For example, binding to proteins such as albumin attenuates the anticoagulant effects in plasma. However, this phenomenon does not explain why rivaroxaban is more potent than apixaban in the plasma-based tests because apixaban exhibits less protein binding than rivaroxaban. Second, in the assays in purified systems, the binding of rivaroxaban and apixaban to factor Xa may differentially alter the kinetics of chromogenic substrate hydrolysis. 44 However, the consistent differences between rivaroxaban and apixaban in all of the tests render this possibility unlikely. Therefore, there appear to be fundamental biochemical differences in the way that rivaroxaban and apixaban interact with factor Xa incorporated within the prothrombinase complex.

In conclusion, we show that rivaroxaban is a more potent inhibitor of prothrombin activation because it inhibits factor Xa incorporated into prothrombinase more rapidly than apixaban. This difference provides an explanation for the observation that rivaroxaban has a greater effect on global tests of coagulation than apixaban.
