# Supplementary material for: Mechanistic Basis for the Differential Effects of Rivaroxaban and Apixaban on Global Tests of Coagulation
Source: TH Open. 2018 May 29;2(2):e190–201. doi: 10.1055/s-0038-1649507 (PMC6524873; doi:10.1055/s-0038-1649507)
Supplement: Supplementary file 1 — Supplementary Material [file 10-1055-s-0038-1649507-s180012.pdf]

Supplementary Materials

Experimental Procedures

Rate Constants of Inhibition

For analysis of thrombin generation, a mixed inhibition model[1] was fit to each time course of AMC production (►**Scheme 1**) and 7 differential equations for the reactants and product were formulated:

$$d[\mathrm{FXa}]/dt = -k_1[\mathrm{FXa}][\mathrm{S}] - k_3[\mathrm{FXa}][\mathrm{I}] + (j_1 + k_2)[\mathrm{FXa} \bullet \mathrm{S}] + j_3[\mathrm{FXa} \bullet \mathrm{I}] \text{ (Eq. 1)}$$

$$d[\mathrm{FXa} \bullet \mathrm{S}]/dt = k_1[\mathrm{FXa}][\mathrm{S}] + j_4[\mathrm{FXa} \bullet \mathrm{S} \bullet \mathrm{I}] - (j_1 + k_2)[\mathrm{FXa} \bullet \mathrm{S}] - k_4[\mathrm{FXa} \bullet \mathrm{S}][\mathrm{I}] \text{ (Eq. 2)}$$

$$d[\mathrm{FXa} \bullet \mathrm{I}]/dt = k_3[\mathrm{FXa}][\mathrm{I}] - j_3[\mathrm{FXa} \bullet \mathrm{I}] \text{ (Eq. 3)}$$

$$d[\mathrm{FXa} \bullet \mathrm{S} \bullet \mathrm{I}]/dt = k_4[\mathrm{FXa} \bullet \mathrm{S}][\mathrm{I}] - j_4[\mathrm{FXa} \bullet \mathrm{S} \bullet \mathrm{I}] \text{ (Eq. 4)}$$

$$d[\mathrm{S}]/dt = -k_1[\mathrm{FXa}][\mathrm{S}] + j_1[\mathrm{FXa} \bullet \mathrm{S}] \text{ (Eq. 5)}$$

$$d[\mathrm{I}]/dt = -k_3[\mathrm{FXa}][\mathrm{I}] + j_3[\mathrm{FXa} \bullet \mathrm{I}] - k_4[\mathrm{FXa} \bullet \mathrm{S}][\mathrm{I}] + j_4[\mathrm{FXa} \bullet \mathrm{S} \bullet \mathrm{I}] \text{ (Eq. 6)}$$

$$d[\mathrm{P}]/dt = k_2[\mathrm{FXa} \bullet \mathrm{S}] \text{ (Eq. 7)}$$

In these equations, FXa is the free factor Xa concentration, S is the concentration of Pefafluor Xa, I is the concentration of inhibitor (apixaban or rivaroxaban), FXa·S is the factor Xa-Pefafluor Xa complex, FXa·I is the factor Xa-inhibitor complex, and FXa·S·I is the ternary factor Xa-Pefafluor Xa-inhibitor complex.

Plots of AMC concentration versus time were analyzed by numerical integration using Berkeley Madonna Software (University of California, Berkeley, CA).[2] The 3-time courses obtained with each inhibitor were fit simultaneously with the assumption that the rate constants k_1, j_1, and k_2 are equal for all of the reactions regardless of whether the inhibitor is present. Therefore, k_1, j_1, and k_2 were fit globally, whereas k_3, j_3, k_4 and j_4 were fit individually. After analysis of the 6 sets of data, the fitted rate constants were averaged. Once the best-fit rate constants were determined, the theoretical dissociation constants for each inhibitor were calculated for free factor Xa ($K_{Xa1} = j_3/k_3$) and for the factor Xa-substrate complex ($K_{Xa2} = j_4/k_4$).

The individual rate constants for association and dissociation of the inhibitors with factor Xa incorporated into the prothrombinase complex were determined by measuring thrombin generation to infer residual factor Xa activity in real-time. Thrombin generation was quantified using DAPA, a probe that specifically inhibits thrombin and exhibits increased fluorescence on doing so,[3] with or without the inhibitors. The time course profiles generated were again fit using a mixed inhibition model[1] (►**Scheme 2**), wherein all forms of factor Xa (E), both free or within prothrombinase, could interact with the inhibitor (I). Prothrombin (P) activation by prothrombinase was limited to the meizothrombin (M) pathway. Ratcheting of meizothrombin, a phenomenon whereby meizothrombin alters its conformation and renders the second cleavage by prothrombinase more effective, was included (k_5, j_5 in ►**Scheme 2**) to be consistent with current models of prothrombinase.[4] The inhibition rate constants for the binding of the inhibitors to prothrombinase when it is in complex with either form of meizothrombin were kept the same. In addition, the fluorescence quantum yield of meizothrombin was taken to be 1.5 times that of thrombin.[5,6] Based on this model, 13 differential equations for the reactants and product were formulated:

$$d[\mathrm{E}]/dt = -k_1[\mathrm{E}][\mathrm{P}] + (j_1 + k_2)[\mathrm{E} \bullet \mathrm{P}] - k_3[\mathrm{E}][\mathrm{M}] + (j_3 + k_4)[\mathrm{E} \bullet \mathrm{M}] - k_6[\mathrm{E}][\mathrm{M2}] + (j_6 + k_7)[\mathrm{E} \bullet \mathrm{M2}] - ki_1[\mathrm{E}][\mathrm{I}] + ji_1[\mathrm{E} \bullet \mathrm{I}] \text{ (Eq. 8)}$$

$$d[\mathrm{E} \bullet \mathrm{P}]/dt = k_1[\mathrm{E}][\mathrm{P}] - (j_1 + k_2)[\mathrm{E} \bullet \mathrm{P}] - ki_2[\mathrm{E} \bullet \mathrm{P}][\mathrm{I}] + ji_2[\mathrm{E} \bullet \mathrm{P} \bullet \mathrm{I}] \text{ (Eq. 9)}$$

$$d[\mathrm{E} \bullet \mathrm{M}]/dt = k_3[\mathrm{E}][\mathrm{M}] - (j_3 + k_4)[\mathrm{E} \bullet \mathrm{M}] - ki_3[\mathrm{E} \bullet \mathrm{M}][\mathrm{I}] + ji_3[\mathrm{E} \bullet \mathrm{M} \bullet \mathrm{I}] \text{ (Eq. 10)}$$

$$d[\mathrm{E} \bullet \mathrm{M2}]/dt = k_6[\mathrm{E}][\mathrm{M2}] - (j_6 + k_7)[\mathrm{E} \bullet \mathrm{M2}] - ki_3[\mathrm{E} \bullet \mathrm{M2}][\mathrm{I}] + ji_3[\mathrm{E} \bullet \mathrm{M2} \bullet \mathrm{I}] \text{ (Eq. 11)}$$

$$d[\mathrm{P}]/dt = -k_1[\mathrm{E}][\mathrm{P}] + j_1[\mathrm{E} \bullet \mathrm{P}] \text{ (Eq. 12)}$$

$$d[\mathrm{M}]/dt = -k_3[\mathrm{E}][\mathrm{M}] + j_3[\mathrm{E} \bullet \mathrm{M}] - k_5[\mathrm{M}] + j_5[\mathrm{M2}] + k_2[\mathrm{E} \bullet \mathrm{P}] \text{ (Eq. 13)}$$

$$d[\mathrm{M2}]/dt = -k_6[\mathrm{E}][\mathrm{M2}] + j_6[\mathrm{E} \bullet \mathrm{M2}] + k_5[\mathrm{M}] - j_5[\mathrm{M2}] \text{ (Eq. 14)}$$

$$d[\mathrm{T}]/dt = k_4[\mathrm{E} \bullet \mathrm{M}] + k_7[\mathrm{E} \bullet \mathrm{M2}] \text{ (Eq. 15)}$$

$$d[\mathrm{I}]/dt = -\{ki_1[\mathrm{E}] + ki_2[\mathrm{E} \bullet P] + ki_3([\mathrm{E} \bullet \mathrm{M}] + [\mathrm{E} \bullet \mathrm{M2}])\}[\mathrm{I}] + ji_1[\mathrm{E} \bullet \mathrm{I}] + ji_2[\mathrm{E} \bullet \mathrm{P} \bullet \mathrm{I}] + ji_3([\mathrm{E} \bullet \mathrm{M} \bullet \mathrm{I}] + [\mathrm{E} \bullet \mathrm{M2} \bullet \mathrm{I}]) \text{ (Eq. 16)}$$

$$d[\mathrm{E} \bullet \mathrm{I}]/dt = ki_1[\mathrm{E}][\mathrm{I}] - ji_1[\mathrm{E} \bullet \mathrm{I}] \text{ (Eq. 17)}$$

$$d[\mathrm{E} \bullet \mathrm{P} \bullet \mathrm{I}]/dt = ki_2[\mathrm{E} \bullet \mathrm{P}][\mathrm{I}] - ji_2[\mathrm{E} \bullet \mathrm{P} \bullet \mathrm{I}] \text{ (Eq. 18)}$$

$$d[\mathrm{E} \bullet \mathrm{M} \bullet \mathrm{I}]/dt = ki_3[\mathrm{E} \bullet \mathrm{M}][\mathrm{I}] - ji_3[\mathrm{E} \bullet \mathrm{M} \bullet \mathrm{I}] \text{ (Eq. 19)}$$

$$d[\mathrm{E} \bullet \mathrm{M2} \bullet \mathrm{I}]/dt = ki_3[\mathrm{E} \bullet \mathrm{M2}][\mathrm{I}] - ji_3[\mathrm{E} \bullet \mathrm{M2} \bullet \mathrm{I}] \text{ (Eq. 20)}$$

The total fluorescence signal (FS) was taken as the sum of both forms of meizothrombin with the coefficient of 1.5 relative to thrombin (T) (Eq. 21).

$$FS = [T] + 1.5([M] + [M2]) \quad \text{(Eq. 21)}$$

Plots of thrombin concentration *versus* time were analyzed by numerical integration with Berkeley Madonna Software (University of California, Berkeley, CA).[2] The data were fit in 4 separate rounds. In the first round, each set of data containing the full range of concentrations of each of the inhibitors was fit individually with little to no imposed restriction. This was repeated 3 times for each inhibitor. The rate constants of the main reaction that do not involve the inhibitor (k_x and j_x) were assumed to be the same. Therefore, during the second round of fitting, the 6 k_x and j_x values estimated from the initial analysis were averaged and allowed to fluctuate by 15% of the mean when fitting the time course in the absence of the inhibitors. Once the best-fit k_x and j_x values for each set of data were obtained (►**Table S1**), they were fixed and used in the third round of fitting. In this round, time course profiles with all concentrations of inhibitor were fit simultaneously with no restrictions imposed on the inhibition rate constants. The 3 sets of ki_x and ji_x values for each inhibitor were then averaged, and in the final round of fitting, were allowed to fluctuate by 15% of the mean. Using the estimated best-fit rate constants, the theoretical dissociation constants for each inhibitor were calculated for prothrombinase without prothrombin ($K_{Pase1} = ji_1/ki_1$), prothrombinase in complex with prothrombin ($K_{Pase2} = ji_2/ki_2$) and for prothrombinase in complex with either form of meizothrombin ($K_{Pase3} = ji_3/ki_3$).

To determine whether the divergent effects of rivaroxaban and apixaban on the coagulation assays are due to differential inhibition of factor Xa leading to differences in substrate recognition, a prothrombin derivative, rMZ, which can only be cleaved at Arg320, was used as the substrate so that initial cleavage at this site could be examined in isolation.[7] rMZ activation by prothrombinase was monitored and the data were analyzed under conditions identical to those used with wild-type prothrombin. The model shown in ►**Scheme 1** was used to describe this reaction, where the substrate (S) was replaced with rMZ and the product (P) was replaced with rMZa. The theoretical dissociation constants for rivaroxaban or apixaban were again calculated using the best-fit rate constants determined as described above.

Table S1 Best-fit rate constants from non-steady state analyses of prothrombin activation by the prothrombinase complex as shown in ►**Scheme 2**

Reaction	Constants	Mean S.D.
E +P⇔E·P	k_1	$(9.32 \pm 1.16) \times 10^{-2}$
	j_1	$(1.38 \pm 0.16) \times 10^{-4}$
P→M	k_2	14.79 ± 1.71
E +M⇔E·M	k_3	$(1.11 \pm 0.15) \times 10^{-3}$
	j_3	$(4.75 \pm 0.69) \times 10^{-6}$
M→T	k_4	1.01 ± 0.13
M⇔M2	k_5	$(4.94 \pm 0.40) \times 10^{-3}$
	j_5	$(1.84 \pm 0.19) \times 10^{-5}$
E +M2⇔E·M2	k_6	$(1.32 \pm 0.13) \times 10^{-1}$
	j_6	$(1.11 \pm 0.13) \times 10^{-2}$
M2→T	k_7	8.14 ± 1.03

The values represent mean ± standard deviation ($n = 6$). The units of the on-rates (k_1, k_3, k_5, and k_6) are $nM^{-1}\cdot second^{-1}$, and off-rates (j_1, j_3, j_5, and j_6) and turnover rates (k_2, k_4, and k_7) are $second^{-1}$.

References

1 Luettgen JM, Knabb RM, He K, Pinto DJ, Rendina AR. Apixaban inhibition of factor Xa: Microscopic rate constants and inhibition mechanism in purified protein systems and in human plasma. J Enzyme Inhib Med Chem 2011;26(04):514–526

2 Kim PY, Nesheim ME. Further evidence for two functional forms of prothrombinase each specific for either of the two prothrombin activation cleavages. J Biol Chem 2007;282(45):32568–32581

3 Nesheim ME, Prendergast FG, Mann KG. Interactions of a fluorescent active-site-directed inhibitor of thrombin: dansylarginine N-(3-ethyl-1,5-pentanediyl)amide. Biochemistry 1979;18(06):996–1003

4 Bianchini EP, Orcutt SJ, Panizzi P, Bock PE, Krishnaswamy S. Ratcheting of the substrate from the zymogen to proteinase conformations directs the sequential cleavage of prothrombin by prothrombinase. Proc Natl Acad Sci U S A 2005;102(29):10099–10104

5 Hibbard LS, Nesheim ME, Mann KG. Progressive development of a thrombin inhibitor binding site. Biochemistry 1982;21(10): 2285–2292

6 Krishnaswamy S, Mann KG, Nesheim ME. The prothrombinase-catalyzed activation of prothrombin proceeds through the intermediate meizothrombin in an ordered, sequential reaction. J Biol Chem 1986;261(19):8977–8984

7 Brufatto N, Nesheim ME. Analysis of the kinetics of prothrombin activation and evidence that two equilibrating forms of prothrombinase are involved in the process. J Biol Chem 2003;278 (09):6755–6764
